# Back to Nature*President’s address before the Medical Association of Central New York, at its twenty-ninth annual meeting, Rochester, N. Y., October 20, 1896.

**Published:** 1896-12

**Authors:** William S. Cheesman

**Affiliations:** Auburn, N. Y.


### BUFFALO MEDICAL JOURNAL.

VOL. XXXVI. DECEMBER, 1896. No. 5.

### Original Communications.

### BACK TO NATURE.*

BY WILLIAM S. CHEESMAN, M. D., Auburn, N. Y.

NORDAU[1] says the upper classes of society are made up largely of "degenerates." That means, of persons who are neurasthenic, hysterical, neurotic. This condition, of which they are for the most part happily unconscious, he traces to causes inherent in modern civilisation—namely, the habitual use of tobacco, alcohol, narcotics; the deteriorating influence of residence in great cities; and the revolutionising of life by new inventions, increased railroad and ocean travel, and the multiplication of books, newspapers, letters, telegrams: in short, to nerve fatigue.

By way of confirming these opinions, Nordau searches modern literature and art for evidences of degeneration. It is there, he finds, that the hysteria of modern life most plainly gives itself away. So the poetry, and painting, and music of the day constitute the material of an unexampled clinic, and I know of nothing more humorous from one point of view than this diagnostic critique, in which the works of modern art, viewed from the novel standpoint of the specialist in nervous disease, are made to yield symptoms and stigmata. Nordau percusses poetry, and gets a cracked-pot sound; he auscultates the opera, and hears an exquisite "bruit du diable;" he puts painting into the circuit, and obtains the reaction of degeneration; and he is shocked by the exaggerated knee-jerk of the stage.

Now, whatever view we may take of this theory, I fancy that many of you, like myself, have felt in reading his learned work that there was much that was suggestive and salutary in its thesis, and have experienced the same lively satisfaction that I did, on realising that inability to understand some modern poets is rather

*President's address before the Medical Association of Central New York, at its twenty-ninth annual meeting, Rochester, N. Y., October 20, 1896.

a credit to one, since their hyperæsthetic and erotic obscurities are, after all, only stigmata of a simple, plain idiocy.

And after reading Nordau one can hardly avoid the question: Have Americans degenerated? We cannot compare ourselves with the early settlers, but I confess to have long believed that as a people we are physically and nervously inferior to descendants of our common ancestry across the sea. I will not use the term "degeneration," for that sounds prognostically hopeless and irreparable. But I will say that as regards sturdiness of physique and nerve stamina, we have deteriorated.

This is rather a common view, and it may be worth while to inquire upon what facts it is based. They are largely impressionistic, and to but a small extent statistical, as is obviously, from the nature of the case, a necessity.

On landing here after a sojourn abroad, where the eye had grown accustomed to the British or German type, I have always been struck by the inferior physical appearance of our people. The men one meets look thin, and pale, and small. This impression depends for its clearness on one's previous habits of observation. To a person accustomed to sizing up men from the athletic standpoint it is vivid; others who take no automatic note of men's build and carriage, it might escape entirely, and they will cavil at my statement. Yet I fancy the most untrained observer would note the difference, could he see on one side of the street such men as throng London, or Edinburgh, or Berlin, and on the other our countrymen.

It has become one of the commonplaces of observation that Americans are increasingly neurotic. Neurasthenia is significantly termed abroad "the American disease." This is the home of "rest cures," and no nation but ours writes and reads treatises on the Technique of Rest. Yet as a race we are incapable of the quietude of old-world life. A contented and continued abode in one place from generation to generation, without variety or change, is to us intolerable. Monotony begets insanity under our skies. The English race has ever shown itself the most enterprising in Europe, and on all the continents and most of the isles of the sea are its dominions. But behind them has stood the quiet, sedate, imperturbable mass of unchanging home life, of which the colonies are but the overflow and surplusage. Are our restlessness and instability tokens of like superabundant energy, or do they spring from the irritability of nervous weakness?

But worse than all, the fertility of our race is falling off. It has long been a matter of general observation that large families of American origin were becoming rare. And statistics seem to bear out this impression. In Europe, generally, the average number of children to each mother is 4.47 (in 1876). Among our foreign-born population here it is in New York state 3.81, and in Providence[2], 3.60. Among American-born mothers it is 3.57 in New York, 3.09 in Providence. The average birth-rate of Massachusetts, Connecticut and Rhode Island[3] in 1888 was 23.9.* In only two civilised countries was the birth-rate as low—namely, Ireland, 22.9, and France, 23.2 ; in the former as a result, universally acknowledged, of grinding poverty, and in the latter of causes engaging the alarmed attention of thoughtful and patriotic Frenchmen.

And this diminution of our birth-rate has occurred in the face of unsurpassed abundance of food and material wealth. Here, if anywhere, has what Malthus termed "the banquet of nature," been lavishly spread before whomsoever would come. So that the general law that the amount of food regulates the increase of population has been plainly evaded here.

Doubtless many factors have united to diminish our fertility. It is a recognised law of economics that the birth-rate varies inversely as the increase of comfort and luxury and the distribution of property. People come to love ease and fear self-sacrifice. For this reason the marriage-rate has fallen steadily during the past twenty years in all the most civilised countries of the world.[5]

But the cause of sterility is not so much the avoidance of marriage as the avoidance of child-bearing after marriage. In France, for instance, the marriage-rate is 7.4 per 1,000 of population, which is not especially low.[6] But the French birth-rate is the lowest in Europe, 21.9 in 1890.[3] French economists explain this by social considerations entirely, such as the desire of keeping a small fortune undivided and of gradually raising the family in social importance. They recall how "social capillarity," the attractive force of a higher social position, acts most strongly in a democracy. Wherever rank is fixed, so that none can rise above his caste, as in monarchial countries, men abandon themselves to careless reproduction. But in a democratic state "each social

*In Providence, R. I., the average number of children born from 1890 to 1895 inclusive was, by American mothers, 3.08; by foreign mothers, 3.54. Providence is selected because of the exactitude of the records kept there.

molecule, having secured the means of existence, struggles to raise itself, impelled by a real force of social attraction." The greater this force, the more sternly are enforced those sacrifices and restrictions which will enable the man to satisfy his ambition. "The birth-rate is, therefore, in inverse ratio to social capillarity."[3]

Yet French writers urge the naturalisation of 50,000 to 100,000 aliens annually in order to improve the reproductive power of the nation,[6] which suggests that their real views of French infertility are more serious than they confess.

Many would explain the infertility of the American democracy in the same way. But if we admit the partial sufficiency of such causes to account for our lessened birth-rate, we cannot forget that sterility of the best stock of a nation, however produced, is a real and great evil, and that it has always been notoriously characteristic of, and contributory to, the decadence of races.

We need not look far to explain any admitted race deterioration in the United States. Man, like other animals, becomes, by long selection and inheritance, adapted to his physical, geographical, social and intellectual evironment. Change this in any respect, and readaptation by eliminations and survivals must ensue. In the first place, ability to resist disease, notably phthisis, is lessened. Pruner states that at Khartoum consumption attacks negro captives and Arabs whenever they lay aside their nomadic habits and live in houses. The same has been observed in regard to our Indians. Icelanders emigrating to Copenhagen suffer at once, and its frequency among the Irish in America has often been noted. And in the second place, a change of life conditions may cause sterility. The breath of civilisation seems to blight the fecundity of savage races ; witness the withering away of the native Australians and some of the South Sea Islanders. Alterations of climate are very unfavorable to fertility and vigor of offspring. Volney states that "neither the Mamalukes, who were a Caucasian race, nor the Turks, who are Mongolians, unless they married native women, which the Mamalukes never did, could continue their race in Egypt, all their offspring perishing in the first or second generation."[7] After much search the late Mr. Twining was unable to find anywhere in India a sample of the third generation from unmixed European stock.

The change from Europe to North America is not comparable in severity to that from Europe to the tropics, but that the climatic conditions here differ radically from those of Europe scarcely needs comparison of temperatures and rainfall.* All who come feel it. English athletes uniformly fail to do their best here, and ruddy immigrants soon lose their vigorous exterior and approach our thin and nervous type.

It seems, therefore, no exaggeration to assert that the American race has not yet so fully adapted itself to our barometric and thermometric vicissitudes, is not yet so thoroughly *acclimated* as to reproduce itself vigorously under civilised conditions, like the parent races across the sea. It is no objection that it settled here 300 years ago, and ought, in all conscience, in that time to feel at home ; for, as a matter of fact, the descendants of the original settlers are so few that they enroll themselves in societies, to emphasise their own regrettable scarcity. The bulk of the race has not lived here over 100 years.

And in that period it has conquered a new empire, competed with those of the old world, and suffered in a greater degree than they all the nervous excitements attending changes of life due to new inventions, of which Nordau makes so much. The result is an intellectually keen, nervously irritable race, devoting too much energy to affairs to have much left for propagation. For "reproductive activity is weakened by every muscular and nervous work,"[3] which is but another statement of the views of Spencer and other economists, that intellectual curtails reproductive energy.

Just here someone will be saying to himself : "This sterility argument must surely be overdone. Think of the families of the Dutchmen and Puritans and pioneers. New conditions did not greatly interfere with their propagating themselves."

I reply : Because they lived close to nature. In any deterioration which we may have suffered, the artificialities of civilisation have borne a part. So that in American nervousness and lessened fruitfulness I read the admonition : Back to nature.

There are three phases of American living contributing to lowered racial vitality, which we as physicians can do something to change :

1. *Neglect of Physical Exercise.*—Excepting the farmers, how much exercise does the average member of society take during a year ? Consider the shop-keepers, clerks, bankers, office-holders, manufacturers, lawyers, deacons, doctors, Sunday-school superin-

---

*But the comparison may be interesting. In 1888, the London temperature range was from 18.4°, the lowest winter, to 87.7°, the highest summer record, while the temperature of New York City was from 0° to 96°.

tendents and their wives, and how many of them have any systematic exercise worthy the name ? Scarcely from one year's end to another do they feel the glow of pulsing blood or the expansion of deep breathing. This they will explain or excuse, but the fact remains. That nerve-weariness, dyspepsia, irritability, misanthropy, are cured by exhilarating out-door activity, they have never found out; and perhaps their physician hasn't told them, since he has never found it out himself ; for, as Bacon has said, "There is a wisdom in this beyond the rules of physic." So they medicate and mope. They ridicule athletic games as boyish. They drive, but never ride. They pay attention to the dinner-bell, not to the dumb-bell, and the club of their acquaintance is not the Indian club, neither is their bar the horizontal bar. In short, it is not uncharitable to say that the lives of the bulk of our middle-aged population are, when viewed from the purely physical standpoint, contemptible. And nature avenges herself in undeveloped limbs, weak hearts, irritable nerves and degenerated blood-vessels. The logical outcome of physical inactivity, nerve-strain, overplus of food, and perhaps wine at dinner, is, in our climate, neurasthenia, or arterio-capillary fibrosis. Nerve exhaustion and early cerebral hemorrhage do a thriving business among the pillars of society and the church.

But a curious fact of history is what may be termed race instinct. After decimating wars, or plagues, or famines, marriage and birth rates rise till the losses are made good. When manners swing dangerously far in any direction, there follows a reaction toward a safer mode. The younger generation are, by virtue of this instinct, betaking themselves to out-door athletics. This "craze" has held its course undeterred by the ridicule of the press, or the fulminations of family doctors. I am not intending a lengthy examination of this movement,* but simply to point out its sociological significance. It is more than a passing fad. It is the intuitive reactionary effort of a new generation after higher physical power. Viewing it from this standpoint, I am sure our profession will be wary of repeating some of the humbug which it has published against athletics in general, and the bicycle for women in particular.

2. *Life in Great Cities.*—One of the prominent movements of the day is the growth of towns. These centers suck in popula-

*Those who care to do so can find one by me in the *Reference Handbook of the Medical Sciences*, Vol. II. Article : Exercise.

tion as a magnet draws iron-filings. Upon the race the effect of this centripetal drift is distinctly injurious. In the first place, overcrowding increases mortality. With such mathematical precision does the death-rate follow density of population, that Dr. Farr constructed a formula by which, the number of inhabitants to a square mile being given, he could predict the mortality. This increased death-tribute of cities is, as we all know, paid largely by children under five. City environment is thus immediately fatal to a piteously large percentage of young children, and how many survive infantile illness to carry its traces in a puny childhood, no tables are prepared to show.

More than all, no statistics can take account of the depressing effect of great city life on the general vitality and nerve tone of children and adults. Its mark on the vicious youth and sad, precocious childhood of the slums is clear; but even in better neighborhoods, who can doubt that for children the noise and hurry and crowd, the devitalised and vitiated air, the monotonous miles of lofty walls and wearying adamant under foot, the crowding of dwellings, the want of playground, induce unhappiness, irritability and nerve wear? Among adults the rush and worry of competition produce the severe neuroses.

But I shall be reminded that one sees no more blooming specimens of childhood, or finer examples of adult health, than are met with on the aristocratic squares of our great towns. The fact, however, is, these people do not really live in their city houses. They sojourn there in winter. Europe, the sea, or the mountains claim them in summer, and southern resorts are peopled by them every spring. It is not city life on such terms that I have in mind.

Yet even for the poor, civilisation seems to be working out relief. Improved communications is the feature of our age. And the question here is one of transportation mainly. If the man can easily and quickly reach his work, cheaper rent, purer air, flowers, fields, and a sky await his family outside. Railroads, the trolley and the bicycle, are bringing us in sight of the day when cities shall be centers for business, learning, pleasure, art, but not for great masses places of abode.

3. *The Postponement of Marriage.*—The Registrar-general's report for 1886 shows a marked difference between the classes of society as to the age at which marriage is contracted.[5] Among miners and artisans men marry at from 24 to 25 years, women from 22 to 23 years, while the professional and independent classes marry on the average five to seven years later in life. Among the more refined and intellectual types there is, therefore, a systematic postponement of marriage.

The explanation is plain. Rich young men are often engrossed in pleasures of a selfish, if not more doubtful, character, and do not settle down until wearied of them. And young professional men with their way to make, have not the means to marry as soon as their wishes might prompt ; while hosts of others decline to marry till they can afford a certain style and standard of establishment—which often ends in their not marrying at all.

The effect of this wilful or enforced delay is doubly disastrous. It robs society of the increase of its most valuable classes during four to seven years of their best physical and reproductive power. As regards numbers and permanence, these classes, therefore, discriminate against themselves. They plot their own extinction by delivering the multiplication of the race over to the lower orders —a duty to which these latter are proverbially faithful. Where universal suffrage rules, this means abdication of government by the people of education and property, and its assumption by low class majorities.

On the individual the effect is also evil. Before an audience of physicians I need do no more than generalise on this point. In our experience bachelorhood means so often irregularity of morals and diseases attendant thereon, or neuroses from sexual suppression ; while late marriages of such individuals are so apt to result in infection and sterility of their wives ; that we, as a profession, hold somber views regarding the salubrity of the single state.

And this impression is justified by Bertillon's statistics, showing that marriage exercises a beneficial influence on the longevity of men and women. According to his tables, the mortality per 1,000 is increasingly heavier with advancing age among the single, till from being four per 1,000 greater at twenty to twenty-four, it becomes fourteen per 1,000 greater at sixty-five to seventy.[5]

Probably now you are asking yourselves : How are doctors to help matters? Can we inject the love of physical activity with a hypodermic syringe, or draw people out of great cities by an aspirator, or remedy the evils of delayed union—when matrimonial—by a surgical operation ? Probably not, but a physician's influence, even his best and greatest, does not end when he signs a prescription, or closes a wound. In the few moments after his duty to the patient is done, and before his visit ends, his highest usefulness may begin. Then his opinion and advice are sought on matters not related directly with sickness.

Then he has his opportunity to mould public opinion. It is in that confidential interval when parents lament the wheel's supposed injuriousness to their daughter's health, or the early entanglement of their son's affections, or when the young man of small means is debating the question of a home—it is then that the doctor can often be of service to his country.

REFERENCES.

1. Entartung.

2. Report of Charles V. Chapin, M D., City Registrar, from whom I have also to acknowledge many kind suggestions.

3. Population and the Social System. F. S. Nitti. Calculated from table, page 173.

4. Achille Guillard. Quoted by Nitti.

5. Article : Vital Statistics Roger S. Tracy. Reference Handbook of the Medical Sciences Vol. IX.

6. Influence of Civilisation on the Movement of Population. P. Leroy Beaulieu. Quoted by Kidd in Social Evolution.

7. Article, Influence of Race and Nationality upon Disease. J. W. Byers. Reference Handbook Medical Sciences. Vol. VIII.

